# Characteristics of ST11 KPC‐2‐producing carbapenem‐resistant hypervirulent *Klebsiella pneumoniae* causing nosocomial infection in a Chinese hospital

**DOI:** 10.1002/jcla.24476

**Published:** 2022-05-06

**Authors:** Pengwen Ouyang, Bin Jiang, Na Peng, Juan Wang, Liang Cai, Yi Wu, Jianrong Ye, Yiping Chen, Hao Yuan, Chaochao Tan, Liming Tan, Liangyi Xie

**Affiliations:** ^1^ Department of Clinical Laboratory Hunan Provincial People’s Hospital (The First Affiliated Hospital of Hunan Normal University) Changsha China; ^2^ Department of Microbiology Laboratory Center for Disease Control and Prevention of Hunan Province Changsha China

**Keywords:** carbapenem resistance, hypervirulence, *Klebsiella pneumoniae*, nosocomial infection

## Abstract

**Background:**

The purpose of our study is to analyze the microbiological and clinical characteristics of carbapenem‐resistant hypervirulent *Klebsiella pneumoniae* (CR‐hvKP) that causes nosocomial infection.

**Methods:**

We collected the carbapenem‐resistant *K*. *pneumoniae* (CRKP) strains that caused nosocomial infection in a hospital in China and collected the relevant clinical data. We characterized these strains for their antimicrobial and virulence‐associated phenotype and genotype and analyzed the clonal relatedness. We screened hypervirulent strains and compared them with non‐hypervirulent strains.

**Results:**

We retrospectively analyzed 62 CRKP strains that caused nosocomial infection in a tertiary hospital within 1 year, of which 41 (41/62, 66.1%) CRKP were considered as CR‐hvKP. All CR‐hvKP strains were multi‐drug resistance (MDR) and the vast majority of isolates (39/41, 95.1%) were ST11 KPC‐2‐producing strains. Two hypermucoviscous isolates and 4 capsular types were found in 41 CR‐hvKP. Twenty‐nine isolates (29/41, 70.7%) showed hypervirulence in *Galleria mellonella* infection model. PFGE showed that ST11‐KL47 CR‐hvKP and ST11‐KL64 CR‐hvKP exhibited a high degree of clonality, while non‐hypervirulent strains were not significant. CR‐hvKP had higher positive rates of *bla*_KPC‐2_ and *bla*_CTX‐M‐65_ and higher levofloxacin resistance (*p* < 0.001, *p* = 0.005 and *p* = 0.046, respectively) when compared to the non‐hypervirulent strains. There was no significant difference between the two groups in terms of in‐hospital mortality (7/41, 17.1% vs 5/21, 23.8%, *p* = 0.743).

**Conclusion:**

Our research finds that ST11 KPC‐2‐producing CR‐hvKP is the main type of CRKP that caused nosocomial infection, and clonal spread has occurred. We provide more information about CR‐hvKP in health care.

## Graphical abstract

Our research finds that ST11 KPC‐2‐producing CR‐hvKP is the main type of CRKP that caused nosocomial infection, which poses a new threat to people's health. And we provide more information about CR‐hvKP in health care.

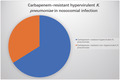

## 1 INTRODUCTION

*Klebsiella pneumoniae* is an important opportunistic pathogen associated with bacterial infections in hospitals. Carbapenem‐resistant *K*. *pneumoniae* (CRKP), which can resist almost all clinically used antibiotics and cause serious infections, poses a huge threat to global public health.^1^, ^2^ *K*. *pneumoniae* sequence type (ST) 258 has contributed significantly to the dissemination of CRKP in the United States and in European countries, but ST11 is predominant in China,^3^ and the genomic characteristics of ST11 *K*. *pneumoniae* strains are significantly different from that of other countries.^4^ The most predominant carbapenemase genes of ST11 CRKP in China are *bla*_KPC‐2_, *bla*_NDM‐1_, *bla*_OXA‐48_, and ST11 KPC‐2‐producing *K*. *pneumoniae* is spreading at an alarming speed.^5^, ^6^, ^7^ ST11 CRKP is emerging in almost all Chinese provinces, and its prevalence in China forces us to conduct better tracing and control of such a pathogen.

Hypervirulent *K*. *pneumoniae* (hvKP) is a more virulent variant of *K*. *pneumoniae*, known for causing invasive infections among young and relatively healthy individuals. This phenomenon is first observed in community infections and mainly occurs in the Asian Pacific Rim.^8^ However, infections caused by hvKP are increasingly being reported worldwide in recent years.^9^, ^10^ Researches find that the genetic determinants of hypervirulence of hvKP are generally on a typical ~200 kb virulence plasmid, as well as integrative conjugal elements.^8^, ^11^ Alarmingly, hvKP can continuously increase antibiotic resistance by acquiring resistance elements.^12^ Convergence of hypervirulent and resistance make hvKP a global pathogen of current concern.^13^

In the past years, reports on carbapenem‐resistant hypervirulent *K*. *pneumoniae* (CR‐hvKP) infection were increasing, even a fatal outbreak caused by CR‐hvKP in a Chinese hospital had been observed.^14^ Worryingly, ST11 CRKP with both hypervirulence and high drug resistance demonstrated limited adaptive cost and enhanced environmental survival,^10^, ^13^, ^15^ which might cause CR‐hvKP to spread more quickly and widely, particularly in the hospital environment.

However, our knowledge of CR‐hvKP is still very limited so far, and more monitoring and research results are urgently needed to fight this pathogen, particularly the pheno‐ and genotypic characteristics of prevalent strains from continually survey in hospitals. This study analyzed the presence of CR‐hvKP among CRKP which caused nosocomial infection in hospitalized patients, and studied the phenotypes and genotypes of the strains, as well as clinical characteristics of the associated patients, which provided more information on understanding CR‐hvKP.

## 2 METHOD

### 2.1 Study design and definition

A one‐year retrospective study was conducted in Hunan Provincial People's Hospital, the First Affiliated Hospital of Hunan Normal University. This study was approved by the ethics committee of Hunan Provincial People's Hospital. The hospital is located in Changsha, Hunan Province, China. It is a large‐scale comprehensive tertiary hospital with nearly 4000 beds. We used VITEK 2 Compact system (bioMérieux, Marcy‐l’Étoile, France) conducting identification and antimicrobial susceptibility test of all strains isolated in Clinical Microbiology Laboratory from January to December 2018. The results of the antimicrobial susceptibility test were interpreted according to the rules provided by the CLSI guidelines.^16^ Strains identified as *K*. *pneumoniae* and resistant to imipenem and/or meropenem (defined as CRKP in this study) were collected. Strains isolated from the same part of the same patient were excluded. Identification results were reconfirmed through VITEK MS (bioMérieux, Marcy‐l’Étoile, France). For strains resistant to imipenem or meropenem, the E test method was used to verify their susceptibility. The antimicrobial susceptibility tests of tigecycline and colistin were carried out by the micro‐broth dilution method. The interpretation of the results was carried out according to the FDA guidelines and EUCAST guidelines, respectively.^17^, ^18^ Multi‐drug resistance (MDR) was defined as resistance to three or more different antimicrobial classes. Disease‐related data of all patients with CRKP infection were collected from medical records, including demographic characteristics, underlying diseases, invasive treatments, infection sites, length of stay, and infection outcome. Nosocomial infection was defined as a new infection which developed 48 h after patient admission. Only patients with a complete hospitalization history and who developed CRKP nosocomial infection were included in the study. CR‐hvKP was defined by the presence of virulence gene *iucA*, *iroN*, *_p_rmpA*, or *_p_rmpA_2_* in CRKP.^14^

### 2.2 Carbapenem‐resistant phenotype and hypermucoviscous phenotype detection

The carbapenem‐resistant phenotype of strains was detected by CLSI recommended modified carbapenem inactivation method (mCIM).^19^ And, the hypermucoviscous phenotype was detected by string test.^14^

### 2.3 Resistance genes, virulence genes, and capsular type genes dection

We detected resistance genes by PCR amplification, including genes encoding carbapenemases (*bla*_KPC_, *bla*_NDM_, *bla*_IMP_, *bla*_VIM_, and *bla*_OXA‐48_) and genes encoding other β‐lactamases (*bla*_CTX‐M_, *bla*_SHV_, and *bla*_TEM_).^20^ We detected virulence genes by PCR amplification, including siderophores (*entB*, *irp*‐*1*, and *irp*‐*2*), fimbriae biosynthesis (*fimH* and *mrkD*), lipopolysaccharide biosynthesis (*wabG*), fucose synthesis (*wcaG*), allantoin metabolism (*allS*), and putative transporter (*peg*‐*344*).^21^, ^22^, ^23^, ^24^ The positive amplification products after agarose gel electrophoresis were sequenced and compared on the NCBI BLAST website (https://blast.ncbi.nlm.nih.gov/blast.cgi). We genotyped the capsular polysaccharide synthesis (*cps*) gene of strains by amplifying and sequencing the *wzi* gene, as previously described.^25^ All primers used in this study are provided in Appendix S1.

### 2.4 Galleria mellonella infection model

Further virulence assessment for CR‐hvKP was conducted by *in vivo G*. *mellonella* infection model.^14^ Healthy *G*. *mellonella* larvae weighing 250–350 mg were obtained from Huiyude Biotech Company (Huiyude Biotech Company, Tianjin, China). Setting up each strain as an experimental group, each experimental group contained 10 larvae. The mid‐log phase culture was diluted with PBS into a bacterial suspension with a concentration of 10^6^ cfu/ml, and 10 μl of the bacterial suspension was injected with a microsyringe into the left forefoot of each larva. After injection, the larvae were kept at 37°C in dark to observe the 72‐h mortality. Strain which the pathogenicity was similar or stronger than the positive control was assessed as hypervirulence *in vivo*. NTUH‐K2044, ZR2 (a non‐hypervirulent *K*. *pneumoniae* strain identified in our previous study), and PBS were used as positive control, negative control, and blank control, respectively.

### 2.5 Multilocus sequence typing (MLST) and pulsed field gel electrophoresis (PFGE)

We conducted MLST by amplifying and sequencing 7 housekeeping genes (*gapA*, *infB*, *mdh*, *pgI*, *phoE*, *rpoB*, and *tonB*).^26^ The allele types and STs were confirmed based on the database provided on the Institut Pasteur website (https://bigsdb.pasteur.fr/). Genetic correlations among the CRKP strains were subsequently analyzed by PFGE.^27^ The PFGE banding pattern was clustered by BioNumerics software version 5.1 (Applied Maths, Kortrijk, Belgium). Isolates with 100% similarity were considered the same PFGE cluster.

### 2.6 Statistic analysis

Statistical analysis was performed using SPSS 19.0 software (IBM Corporation, Armonk, NY, USA). Continuous variables were assessed by Student's *t* tests. The comparison of the categorical variables was calculated by Chi‐square test or Fisher's exact test. *p* value <0.05 was considered to be statistically significant.

## 3 RESULTS

### 3.1 Strain and patient information

**FIGURE 1:**
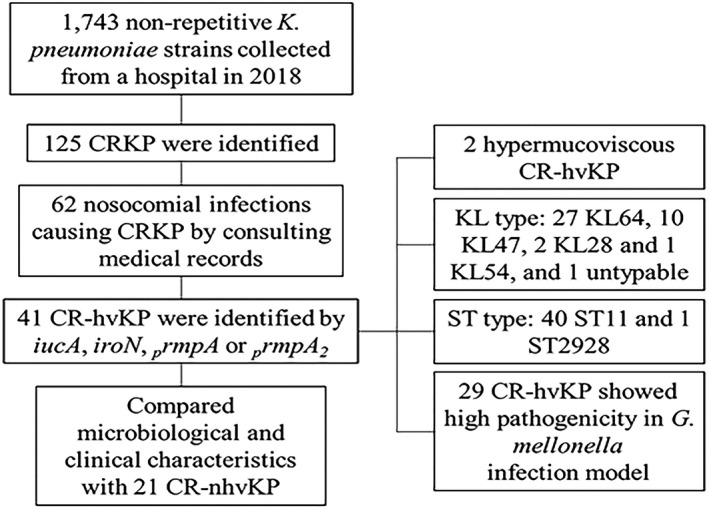
Routine monitoring of K. pneumoniae and research process of this study

In this study, a total of 1743 *K*. *pneumoniae* strains from Hunan Provincial People's Hospital were collected between January 1 and December 31, 2018, by routine monitoring, among which 125 (7.17%) CRKP were identified (Figure 1). By consulting medical records, 62 CRKP causing nosocomial infections from 62 patients were confirmed, including 36 (58.1%) in surgical department, 19 (30.6%) in ICU, 5 (8.1%) in internal medicine department, and 2 (3.2%) in pediatric department. CRKP strains were isolated from a variety of sample types. Samples of respiratory tract and bile accounted for the largest proportion, with 25 (40.3%) cases and 11 (17.7%) cases, respectively. The others were 9 (14.5%) ascites cases, 8 (12.9%) blood cases, 4 (6.5%) wound cases, 3 (4.9%) pus cases, 1 (1.6%) reproductive tract case, and 1(1.6%) urine case. No pyogenic liver abscess or metastatic infection was reported in the 62 CRKP‐infected patients.

**FIGURE 2:**
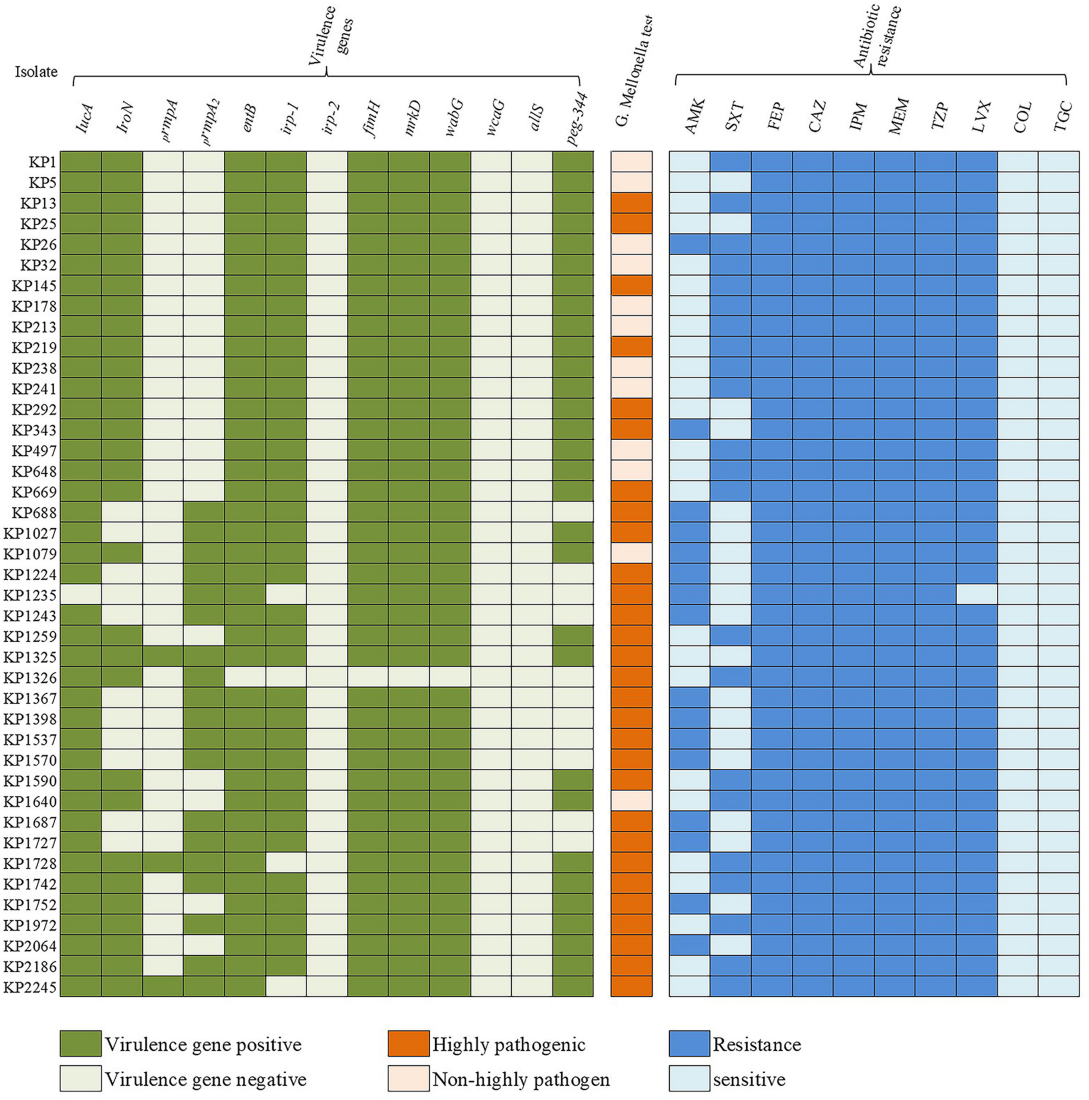
Carriage of virulence genes, in vivo virulence and antimicrobial resistance features of 41 CR‐hvKP strains. AMK, amikacin; SXT, trimethoprim/sulfamethoxazole; FEP, cefepime; CAZ, ceftazidime; IPM, imipenem; MEM, meropenem; TZP, piperacillin/Tazobactam; LVX, levofloxacin; COL, colistin; TGC, tigecycline

More than half of the 62 (41/62, 66.1%) CRKP were CR‐hvKP. The positive rates of *iucA*, *iroN*, *_p_rmpA*, and *_p_rmpA_2_* in 41 CR‐hvKP were 40/41 (97.6%), 30/41 (73.2%), 3/41 (7.3%), and 19/41 (46.3%), respectively, among which 3 isolates had all four virulence genes (Figure 2). CR‐hvKP mainly came from respiratory tract specimens (14/41, 34.2%). Males were mostly infected (30/41, 73.2%). Surgery department and ICU were the main epidemic departments (25/41, 61.0% and 12/41, 29.3%, respectively). Only 7 cases of CR‐hvKP infection caused fatal outcomes (7/41, 17.1% mortality rate) (Table 1). We compared the clinical features between CR‐hvKP and carbapenem‐resistant non‐hypervirulent *K*. *pneumoniae* (CR‐nhvKP). Through our analysis, there were no significant differences between the two groups in terms of age, gender, underlying diseases, invasive treatments, infection sites, length of hospital stay, and in‐hospital mortality (Table 2).

**TABLE 1:** Clinical characteristics of 41 CR‐hvKP strains

Strain ID	Source	Department	Collection date	Outcome	mCIM	Antibiotic resistance genes	String test	Capsular type	ST
KP1	Reproductive tract	SSD	2018/3/7	Survived	+	*bla*_KPC‐2_, *bla*_SHV‐11_, *bla*_TEM‐1C_	−	KL64(*wzi*64)	11
KP5	Blood	GSD	2018/3/1	Survived	+	*bla*_KPC‐2_, *bla*_SHV‐11_	−	KL64(*wzi*64)	11
KP13	Sputum	RD	2018/3/12	Death	+	*bla*_KPC‐2_, *bla*_SHV‐11_	−	KL64(*wzi*64)	11
KP25	Ascites	GSD	2018/1/5	Survived	+	*bla*_KPC‐2_, *bla*_SHV‐11_	−	KL64(*wzi*64)	11
KP26	Wound	SSD	2018/1/6	Survived	+	*bla*_KPC‐2_, *bla*_SHV‐11_, *bla*_TEM‐1_	−	KL64(*wzi*64)	11
KP32	Blood	GSD	2018/3/7	Survived	+	*bla*_KPC‐2_, *bla*_SHV‐12_	−	KL64(*wzi*64)	11
KP145	Bile	GSD	2018/3/31	Survived	+	*bla*_KPC‐2_, *bla*_SHV‐11_	−	KL64(*wzi*64)	11
KP178	Blood	ICU	2018/5/14	Death	+	*bla*_KPC‐2_, *bla*_SHV‐11_	−	KL64(*wzi*64)	11
KP213	Blood	ICU	2018/4/17	Death	+	*bla*_KPC‐2_, *bla*_SHV‐11_	−	KL64(*wzi*64)	11
KP219	Blood	GSD	2018/4/8	Survived	+	*bla*_KPC‐2_, *bla*_SHV‐11_	+	KL64(*wzi*64)	11
KP238	Ascites	HSD	2018/4/17	Survived	+	*bla*_KPC‐2_, *bla*_SHV‐11_	−	KL64(*wzi*64)	11
KP241	Sputum	ICU	2018/4/19	Survived	+	*bla*_KPC‐2_, *bla*_SHV‐12_	−	KL64(*wzi*64)	11
KP292	Ascites	GSD	2018/4/25	Survived	+	*bla*_KPC‐2_, *bla*_SHV‐12_	−	KL64(*wzi*64)	11
KP343	Ascites	HSD	2018/5/8	Survived	+	*bla*_KPC‐2_, *bla*_CTX‐M‐65_, *bla*_SHV‐11_, *bla*_TEM‐1_	−	KL64(*wzi*64)	11
KP497	Sputum	GSD	2018/6/11	Survived	+	*bla*_KPC‐2_, *bla*_SHV‐12_	−	Untypable	11
KP648	Urine	ICU	2018/6/26	Survived	+	*bla*_KPC‐2_, *bla*_SHV‐12_	−	KL64(*wzi*64)	11
KP669	Sputum	ICU	2018/6/26	Survived	+	*bla*_KPC‐2_, *bla*_SHV‐11_	−	KL64(*wzi*64)	11
KP688	Bile	HSD	2018/6/29	Survived	+	*bla*_KPC‐2_, *bla*_CTX‐M‐65_, *bla*_SHV‐11_	−	KL47(*wzi*209)	11
KP1027	Blood	HSD	2018/8/2	Survived	+	*bla*_KPC‐2_, *bla*_CTX‐M‐65_, *bla*_SHV‐11_	−	KL47(*wzi*209)	11
KP1079	Bile	HSD	2018/8/4	Survived	+	*bla*_KPC‐2_, *bla*_SHV‐12_, *bla*_TEM‐1_	−	KL64(*wzi*64)	11
KP1224	Sputum	ICU	2018/8/14	Survived	+	*bla*_KPC‐2_, *bla*_CTX‐M‐65_, *bla*_SHV‐11_	−	KL47(*wzi*209)	11
KP1235	Sputum	RD	2018/8/15	Survived	+	*bla*_IMP‐4_, *bla*_CTX‐M‐3_, *bla*_SHV‐187_, *bla*_TEM‐1_	−	KL54(*wzi*115)	2928
KP1243	Sputum	ICU	2018/8/15	Survived	+	*bla*_KPC‐2_, *bla*_CTX‐M‐65_, *bla*_SHV‐11_	−	KL47(*wzi*209)	11
KP1259	Blood	HSD	2018/8/19	Death	+	*bla*_SHV‐11_	−	KL64(*wzi*64)	11
KP1325	Ascites	HSD	2018/8/25	Survived	+	*bla*_KPC‐2_, *bla*_CTX‐M‐65_, *bla*_SHV‐12_	−	KL64(*wzi*64)	11
KP1326	Sputum	HSD	2018/8/26	Survived	+	*bla*_KPC‐2_, *bla*_TEM‐1_	−	KL64(*wzi*64)	11
KP1367	Pus	GSD	2018/8/29	Survived	+	*bla*_KPC‐2_, *bla*_CTX‐M‐65_, *bla*_SHV‐11_	−	KL47(*wzi*209)	11
KP1398	Sputum	NSD	2018/8/14	Death	+	*bla*_KPC‐2_, *bla*_CTX‐M‐65_, *bla*_SHV‐11_	−	KL47(*wzi*209)	11
KP1537	Wound	GSD	2018/9/5	Death	+	*bla*_KPC‐2_, *bla*_CTX‐M‐65_, *bla*_SHV‐11_	−	KL47(*wzi*209)	11
KP1570	Ascites	HSD	2018/9/6	Survived	+	*bla*_KPC‐2_, *bla*_CTX‐M‐65_, *bla*_SHV‐11_	−	KL47(*wzi*209)	11
KP1590	Pus	HSD	2018/9/9	Survived	+	*bla*_KPC‐2_, *bla*_SHV‐11_	−	KL64(*wzi*64)	11
KP1640	Sputum	ICU	2018/9/13	Survived	+	*bla*_KPC‐2_, *bla*_SHV‐11_	−	KL64(*wzi*64)	11
KP1687	Sputum	NSD	2018/9/16	Death	+	*bla*_KPC‐2_, *bla*_CTX‐M‐65_, *bla*_SHV‐11_	−	KL47(*wzi*209)	11
KP1727	Wound	ICU	2018/9/20	Survived	+	*bla*_KPC‐2_, *bla*_CTX‐M‐65_, *bla*_SHV‐11_	−	KL47(*wzi*209)	11
KP1728	Sputum	ICU	2018/9/21	Survived	+	*bla*_KPC‐2_, *bla*_CTX‐M‐65_, *bla*_SHV‐11_	−	KL28(*wzi*84)	11
KP1742	Ascites	HSD	2018/9/23	Survived	+	*bla*_KPC‐2_, *bla*_SHV‐12_	−	KL64(*wzi*64)	11
KP1752	Ascites	HSD	2018/9/25	Survived	+	*bla*_KPC‐2_, *bla*_CTX‐M‐65_, *bla*_SHV‐12_, *bla*_TEM‐1_	−	KL64(*wzi*64)	11
KP1972	Sputum	ND	2018/10/15	Survived	+	*bla*_KPC‐2_, *bla*_SHV‐12_	+	KL64(*wzi*64)	11
KP2064	Bile	ICU	2018/11/5	Survived	+	*bla*_KPC‐2_, *bla*_CTX‐M‐65_, *bla*_SHV‐11_, *bla*_TEM‐1_	−	KL64(*wzi*64)	11
KP2186	Bile	HSD	2018/12/6	Survived	+	*bla*_KPC‐2_, *bla*_SHV‐11_	−	KL64(*wzi*64)	11
KP2245	Sputum	ICU	2018/12/23	Survived	+	*bla*_KPC‐2_, *bla*_CTX‐M‐65_, *bla*_SHV‐11_	−	KL28(*wzi*84)	11

Abbreviations: SSD, Spinal Surgery Department; GSD, General Surgery Department; RD, Respiratory Department; ICU, Intensive Care Unit; HSD, Hepatobiliary Surgery Department; NSD, Neurological Surgery Department; ND, Nephrology Department.

**TABLE 2:** Comparison of clinical features between CR‐hvKP strains and CR‐nhvKP strains

Variables	Total (*n* = 62)	CR‐hvKP (*n*=41)	CR‐nhvKP (*n*=21)	χ^2^	*p* value
Demographics					
Age(mean ± SD)	56.9 ± 19.9	57.07 ± 18.14	56.86 ± 23.50	NA^a^	0.923
Age (<1)	2 (3.2%)	0 (0%)	2 (9.5%)	0.698^b^	0.403
Age (1–49)	17 (27.4%)	14 (34.1%)	3 (14.3%)		
Age (≥50)	43 (69.4%)	27 (65.9%)	16 (76.2%)		
Male	41 (66.1%)	30 (73.2%)	11 (52.4%)	2.680	0.102
Underlying diseases					
Diabetes mellitus	8 (12.9%)	7 (17.1%)	1 (4.8%)	NA	0.247
Chronic cardiovascular system diseases	6 (9.7%)	2 (4.9%)	4 (19.0%)	NA	0.167
Chronic respiratory system diseases	4 (6.5%)	1 (2.4%)	3 (14.3%)	NA	0.108
Tumor	7 (11.3%)	3 (7.3%)	4 (19.0%)	NA	0.214
Chronic kidney diseases	1 (1.6%)	1 (2.4%)	0 (0%)	NA	1.000
Chronic Hepatitis/cirrhosis	4 (6.5%)	3 (7.3%)	1 (4.8%)	NA	1.000
Invasive treatments					
Puncture or venous catheterization	30 (48.4%)	19 (46.3%)	11 (52.4%)	0.203	0.652
Mechanical Ventilation	18 (29.0%)	13 (31.7%)	5 (23.8%)	0.420	0.517
History of surgery	38 (61.3%)	27 (65.9%)	11 (52.4%)	1.062	0.303
Infection sites					
Respiratory tract	25 (40.3%)	14 (34.2%)	11 (52.4%)	1.919	0.166
Biliary tract	11 (17.7%)	5 (12.2%)	6 (28.6%)	NA	0.160
Abdomen	9 (14.5%)	8 (19.5%)	1 (4.8%)	NA	0.150
Blood	8 (12.9%)	7 (17.1%)	1 (4.8%)	NA	0.247
Wound	4 (6.5%)	3 (7.3%)	1 (4.8%)	NA	1.000
Pus	3 (4.9%)	2 (4.9%)	1 (4.8%)	NA	1.000
Urinary tract	1 (1.6%)	1 (2.4%)	0 (0%)	NA	1.000
Reproductive tract	1 (1.6%)	1 (2.4%)	0 (0%)	NA	1.000
Hospitalization					
<30 days	51 (82.3%)	31 (75.6%)	20 (95.2%)	1.052	0.305
≥30 days	11 (17.7%)	10 (24.4%)	1 (4.8%)	NA	0.080
Outcome					
In‐hospital mortality	12 (19.4%)	7 (17.1%)	5 (23.8%)	0.404	0.525

Note

NA: Data not applicable.

^a^ Assessed by Student's *t* tests.

^b^ For the number of patients younger than 1 year old is too small, it is combined with "Age (1–49)" in statistical analysis.

### 3.2 Antibiotic resistance characteristics of CR‐hvKP compared to CR‐nhvKP in nosocomial infection

All 62 CRKP were MDR. The 62 CRKP had high drug resistance rates for most of the antibacterial drugs that were tested, except for colistin and tigecycline. For CR‐hvKP, the resistance levels for cefepime, ceftazidime, imipenem, meropenem, and piperacillin/tazobactam were all 100%, followed by levofloxacin (97.6%), trimethoprim/sulfamethoxazole (51.2%), amikacin (41.5%), colistin (0%), and tigecycline (0%) (Figure 2, Table 3). In terms of resistance rate for levofloxacin from the CR‐hvKP group, it was significantly higher than those from the CR‐nhvKP group (97.6% vs 52.4%, *p* < 0.001) (Table 3).

**TABLE 3:** Comparison of resistance features between CR‐hvKP and CR‐nhvKP

Variables	Total (*n* = 62)	CR‐hvKP (*n* = 41)	CR‐nhvKP (*n* = 21)	χ^2^	P value
Antibiotic resistance rates					
AMK	24 (38.7%)	17 (41.5%)	7 (33.3%)	0.387	0.534
SXT	34 (54.8%)	21 (51.2%)	13 (61.9%)	0.640	0.424
FEP	62 (100.0%)	41 (100.0%)	21 (100.0%)	NA	NA
CAZ	62 (100.0%)	41 (100.0%)	21 (100.0%)	NA	NA
IPM	62 (100.0%)	41 (100.0%)	21 (100.0%)	NA	NA
MEM	62 (100.0%)	41 (100.0%)	21 (100.0%)	NA	NA
TZP	61 (98.4%)	41 (100.0%)	20 (95.2%)	NA	0.339
LVX	52 (83.9%)	40 (97.6%)	11 (52.4%)	NA	<0.001
COL	0 (0%)	0 (0%)	0 (0%)	NA	NA
TGC	0 (0%)	0 (0%)	0 (0%)	NA	NA
Antibiotic‐resistant genes					
*bla*_KPC‐2_	49 (79.0%)	39 (95.1%)	10 (47.6%)	NA	<0.001
*bla*_IMP‐4_	1 (1.6%)	1 (2.4%)	0 (0%)	NA	1.000
*bla*_CTX‐M‐3_	3 (4.8%)	1 (2.4%)	2 (9.5%)	NA	0.263
*bla*_CTX‐M‐14_	1 (1.6%)	0 (0%)	1 (4.8%)	NA	0.339
*bla*_CTX‐M‐15_	1 (1.6%)	0 (0%)	1 (4.8%)	NA	0.339
*bla*_CTX‐M‐65_	19 (30.6%)	16 (39.0%)	3 (14.3%)	3.999	0.046
*bla*_SHV‐1_	2 (3.2%)	0 (0%)	2 (9.5%)	NA	0.111
*bla*_SHV‐11_	40 (64.5%)	29 (70.7%)	11 (52.4%)	2.043	0.153
*bla*_SHV‐12_	11 (17.7%)	10 (24.4%)	1 (4.8%)	NA	0.080
*bla*_SHV‐28_	1 (1.6%)	0 (0%)	1 (4.8%)	NA	0.339
*bla*_SHV‐187_	1 (1.6%)	1 (2.4%)	0 (0%)	NA	1.000
*bla*_TEM‐1_	17 (27.4%)	8 (19.5%)	9 (42.9%)	3.803	0.051

Abbreviations: AMK, amikacin; SXT, trimethoprim/sulfamethoxazole; FEP, cefepime; CAZ, ceftazidime; IPM, imipenem; MEM, meropenem; TZP, piperacillin/tazobactam; LVX, levofloxacin; COL, colistin; TGC, tigecycline; NA, Data not applicable.

The mCIM tests of all 62 CRKP strains were positive. Two families of carbapenemase genes were identified from 62 CRKP strains: *bla*_KPC‐2_ (49/62, 79.0%) and *bla*_IMP‐4_ (1/62, 1.6%). Most (39/41, 95.1%) CR‐hvKP harbored *bla*_KPC‐2_, and 1 (1/41, 2.4%) harbored *bla*_IMP‐4_. 1 CR‐hvKP isolate did not carry *bla*_KPC_, *bla*_NDM_, *bla*_IMP_, *bla*_VIM_, or *bla*_OXA‐48_, but the mCIM phenotype test was positive, indicating that it might harbor other carbapenem resistance genes. Other β‐lactamase genes were detected in CR‐hvKP strains, including *bla*_CTX‐M‐3_, *bla*_CTX‐M‐15_, *bla*_CTX‐M‐65_, *bla*_SHV‐11_, *bla*_SHV‐12_, and *bla*_TEM‐1_. The positive rates of *bla*_KPC‐2_ and *bla*_CTX‐M‐65_ in CR‐hvKP group were both higher than that of CR‐nhvKP group (*p* < 0.001 and *p* = 0.046, respectively) (Table 3).

### 3.3 Virulence‐related characteristics of CR‐hvKP compared with CR‐nhvKP in nosocomial infection

By string test, only 2/62 (3.2%) CRKP strains were hypermucoviscous (both were CR‐hvKP). *wzi* sequencing revealed 11 capsular types in 62 CRKP (28 KL64, 17 KL47, 2 KL28, 1 KL54, 1 KL136, 1 KL38, 1 KL19, 1 KL17, 1 KL123, 1 KL23, and 1 KL25, with 7 untypable). The vast majority of CR‐hvKP belonged to KL64 and KL47, and KL64 was the dominant capsular type (27/41 KL64, 10/41 KL47, 2/41 KL28, and 1/41 KL54, with 1 untypable), while in the CR‐nhvKP group more diverse capsular types were found. Capsule type KL64 (27/41 vs 1/21, *p* < 0.001) was significantly associated with the CR‐hvKP group when compared to the CR‐nhvKP group (Table 4). For virulence background genes in 62 CRKP strains, the positive rates of *fimH*, *wabG*, *entB*, *mrkD*, *irp*‐*1*, *peg*‐*344*, *irp*‐*2*, *wcaG*, and *allS* were 96.8%, 95.2%, 93.5%, 93.5%, 77.4%, 58.1%, 0%, 0%, and 0%, respectively. Virulence genes *irp*‐*1* (37/41 vs 11/21, *p* = 0.003) and *peg*‐*344* (30/41 vs 6/21, *p*=0.001) were significantly associated with the CR‐hvKP group when compared to the CR‐nhvKP group (Table 4). By *G*. *mellonella* infection model, we found that 29/41 (70.7%) CR‐hvKP strains showed hypervirulence *in vivo* (Figure 2).

**TABLE 4:** Comparison of bacterial characteristics between CR‐hvKP and CR‐nhvKP

Variables	Total (*n* = 62)	CR‐hvKP (*n* = 41)	CR‐nhvKP (*n* = 21)	χ^2^	P value
Hypermucoviscosity	2 (3.2%)	2 (4.9%)	0 (0%)	NA	0.545
Capsular types					
KL64	28 (45.2%)	27 (65.9%)	1 (4.8%)	20.928	<0.001
KL47	17 (27.4%)	10 (24.4%)	7 (33.3%)	0.558	0.455
KL28	2 (3.2%)	2 (4.9%)	0 (0%)	NA	0.545
KL54	1 (1.6%)	1 (2.4%)	0 (0%)	NA	1.000
Other capsular type	14 (22.6%)	1 (2.4%)	13 (61.9%)	NA	<0.001
MLST					
ST11	49 (79.0%)	40 (97.6%)	9 (42.9%)	NA	<0.001
ST2928	1 (1.6%)	1 (2.4%)	0 (0%)	NA	1.000
Other ST	12 (19.4%)	0 (0%)	12 (57.1%)	NA	<0.001
Siderophores					
*entB*	58 (93.5%)	40 (97.6%)	18 (85.7%)	NA	0.108
*irp‐1*	48 (77.4%)	37 (90.2%)	11 (52.4%)	NA	0.003
*irp‐2*	0 (0%)	0 (0%)	0 (0%)	NA	NA
Adhesin					
*fimH*	60 (96.8%)	40 (97.6%)	20 (95.2%)	NA	1.000
*mrkD*	58 (93.5%)	40 (97.6%)	18 (85.7%)	NA	0.108
Lipopolysaccharide biosynthesis					
*wabG*	59 (95.2%)	40 (97.6%)	19 (90.5%)	NA	0.263
Fucose synthesis					
*wcaG*	0 (0%)	0 (0%)	0 (0%)	NA	NA
Allantoin metabolism					
*allS*	0 (0%)	0 (0%)	0 (0%)	NA	NA
Putative transporter					
*peg‐344*	36 (58.1%)	30 (73.2%)	6 (28.6%)	11.344	0.001

Note

NA, Data not applicable.

### 3.4 Phylogenetic characteristics of CR‐hvKP isolates

A total of 13 STs among 62 CRKP were identified in the study. ST11 was the most prevalent ST (49/62), followed by ST571 (2/62), ST15 (1/62), ST37 (1/62), ST70 (1/62), ST101 (1/62), ST414 (1/62), ST515 (1/62), ST1040 (1/62), ST1779 (1/62,), ST1933 (1/62), ST2928 (1/62), and new ST (1/62). The majority of CR‐hvKP belonged to ST11 (40/41, 97.6%) and 1 belonged to ST2928 (Table 1). More scattered clones were observed in the CR‐nhvKP group. ST11 (40/41 vs 9/21, *p* < 0.001) was strongly associated with CR‐hvKP when compared to CR‐nhvKP (Table 4).

In the PFGE test, 1 CRKP strain had no complete bands despite several repeats. 61 CRKP strains displayed 36 PFGE clusters (Appendix S2). Clusters 10, 15, and 18 were the three main clusters, which all consisting of CR‐hvKP strains. Cluster 10 included 7 strains that all belonged to ST11‐KL47, cluster 15 included 11 strains that all belonged to ST11‐KL64, and cluster 18 included 8 strains that all belonged to ST11, among which 7 were KL64. Notably, all 7 CR‐hvKP strains in cluster 10 shared the same virulence genes and resistance genes, as well as high lethality in the *G*. *mellonella* infection model and resistance pattern. The 7 strains were all isolated between June and September 2018, suggesting clonal transmission. For CR‐nhvKP, the homology of isolates was more diverse; only one cluster included 2 strains with 100% similarity was found.

## 4 DISCUSSION

Hypervirulent *K*. *pneumoniae* is originally known for community infections and has become a global clinical problem. This study analyzed the CR‐hvKP that caused nosocomial infection during a specific period in a Chinese hospital, and we provided more medical institution related data about the “superbug.”

By detecting virulence genes highly related to hypervirulence, we found 66.1% CR‐hvKP from 62 CRKP strains. We further investigated the virulence genetic background of these strains, and we found 70.7% CR‐hvKP showed hypervirulence in *G*. *mellonella* infection model. Our data showed that the hypervirulent *K*. *pneumoniae* with carbapenem resistance was the main member of the CRKP that caused nosocomial infection in hospitalized patients and clonal spread of this pathogen had occurred. To date, reports on nosocomial infection CR‐hvKP are very limited. Liu et al^28^ found 45.3% hypervirulent *K*. *pneumoniae* strains from 117 cases of nosocomial *K*. *pneumoniae* infection, reporting a substantial change in the epidemiology of hypervirulent *K*. *pneumoniae*. Yang et al. found that CRKP strains harbored a virulence‐encoding plasmid accounted for 58% of 784 *bla*_KPC‐2_‐bearing CRKP strains in a recently published study in China.^29^ Our data partially supported this trend.

These CR‐hvKP were mainly isolated from respiratory tract specimens, and most of the cases occurred in surgical department and ICU. These findings were partly consistent with the results of previous studies.^10^ Zhao et al^30^ found that hypervirulent *K*. *pneumoniae* strains were prevalent in surgical site infections, with an infection rate as high as 46.0%, suggesting surgical patients should be considered to take more stringent infection prevention measures. In previous studies on hypervirulent *K*. *pneumoniae* infection, it was found that males were more commonly infected, and those in the fifth and sixth decades of life were of the highest risk.^8^ In our study, we did not find similar results. It is worth noting that we had observed CRKP infections in hospitalized infants. Although the strains were non‐hypervirulent, it releases a danger signal, as Wang et al.^31^ reported. Metastatic spreading or pyogenic liver abscess are typical clinical features of hypervirulent *K*. *pneumoniae* infection, but in our research, we did not find such performances.

No significant differences were found in age, gender, underlying diseases, invasive treatments, infection sites, hospitalization time, and mortality rates between hypervirulent group and non‐hypervirulent group. From these existing data, it is difficult to find the serious impact of hypervirulence. These results could be explained from the following two aspects. Firstly, patients were in the period of medical observation when the infection occurred. Due to timely intervention, the infections were prevented from worsening. Secondly, these hypervirulent strains might lose some virulence factors, which reduced their pathogenicity to host.

All CR‐hvKP strains were MDR in this study. Of all carbapenemase genes detected, gene *bla*_KPC‐2_ showed the highest detection rate, which is the most common type of carbapenemase gene carried by ST11 CR‐hvKP in China.^10^, ^14^, ^32^ And, we found that *bla*_KPC‐2_ and *bla*_CTX‐M‐65_ tended to exist in hypervirulent strains. Hypervirulent strains were more resistant to levofloxacin, which may result from the co‐transfer of *bla*_KPC_ and quinolone resistance factors. For *bla*_KPC_ is usually carried by plasmid, the plasmid often simultaneously carries other drug resistance genes.^11^ In addition, all strains in this research showed a high level of susceptibility to colistin and tigecycline. Continuous resistance screening is still necessary.

Obviously, we reported a lower incidence of hypermucoviscosity phenotype of CR‐hvKP in this study. Two hypermucoviscous strains were from blood and sputum; both showed high lethality in the *G*. *mellonella* infection model. Gene *_p_rmpA_2_* was detected in hypermucoviscous strain KP1972, which is consistent with the finding that *_p_rmpA_2_* is responsible for hypermucoviscosity.^33^ However, we observed a strain (KP219) with hypermucoviscosity but lack of *_p_rmpA* and *_p_rmpA_2_*, as well as strains carrying *_p_rmpA* and/or *_p_rmpA_2_* but not hypermucoviscous. These cases are less common in hypervirulent *K*. *pneumoniae*, but both have been reported.^9^, ^34^ This phenomenon deserves further investigation.

Through MLST and capsular type detection, we found that 41 CR‐hvKP in this study could mainly be divided into two series: 27 ST11‐KL64 isolates and 10 ST11‐KL47 isolates. All 27 ST11‐KL64 CR‐hvKP were positive for a combination of *iucA*‐*iroN* genes (with a few strains that also carrying *_p_rmpA* and/or *_p_rmpA_2_*), while all 10 ST11‐KL47 CR‐hvKP were positive for a combination of *iucA*‐ *_p_rmpA_2_* genes, suggesting that two different developmental series might have formed. This was confirmed by PFGE analysis. 7 ST11‐KL47 CR‐hvKP which from the PFGE cluster 10 shared the same virulence and resistance characteristics and all isolated from a short period of time, suggested that clonal spread had occurred in our hospital. Interestingly, among ST11‐KL64 CR‐hvKP in PFGE cluster 15 and 18, no consistent characteristics were observed. This might result from the rapid reshaping and diversification of the genome pool of ST11‐KL64 CR‐hvKP.^35^ Moreover, we found 2 ST11‐KL28 KPC‐2‐producing CR‐hvKP and 1 ST2928‐KL54 IMP‐4‐producing CR‐hvKP. As far as we know, hvKP of these types had not been reported, suggesting the diversity of CR‐hvKP evolution.

In this study, only 3 strains with all the four virulence genes positive were detected; the three all showed high lethality in the *G*. *mellonella* infection model, suggesting complete pLVPK plasmid harboring. Interestingly, KP13, KP145, KP669, and KP1590 shared the same drug resistance type and genotype with KP178, KP213, KP238, and KP1640, but they showed opposite results in the *G*. *mellonella* infection model. This might be caused by the presence of undetected virulence factors. In addition, we also found that virulence genes *irp*‐*1* and *peg*‐*344* mainly existed in hypervirulent strains, indicating that these two genes may contribute to the virulence. In future research, the mechanism of hypervirulence is still worthy of attention.

This study only analyzed CRKP isolated from one hospital in China in one year. The sample size is very limited, and larger‐scale or multi‐center research results are needed to support the above results. In addition, the condition for screening hypervirulent strains in this study is the presence of one or more of virulence gene markers, rather than virulence plasmid, which may cause an overestimation of CR‐hvKP. And, we cannot provide data related to plasmid analysis. Given the excellent ability of *K*. *pneumoniae* to obtain exogenous genes encoding resistance and virulence, we need to arouse sufficient vigilance over CR‐hvKP.

## 5 CONCLUSION

ST11 KPC‐2‐producing CR‐hvKP is widespread in our hospital, and two significant lineages of ST11‐KL64 and ST11‐KL47 have emerged and clonal transmission occurred. It is urgent to develop novel rapid and convenient methods to accurately distinguish hypervirulent *K*. *pneumoniae* and classical *K*. *pneumoniae* in order to perform precise treatment and deal with the spread of CR‐hvKP.

## AUTHOR CONTRIBUTIONS

LX and PO conceived and designed the study. PO, BJ, NP, and JW contributed to data collection and experiments implementation. PO, LC, YW, JY, YC, HY, CT, and LT contributed to data analysis and data interpretation. PO and LX contributed to writing the report and revising the report. All authors read and approved the final version of the manuscript.

## CONFLICT OF INTEREST

All authors‐none to declare.

## Supporting information

Appendix S1

Appendix S2

## Data Availability

The data that support the findings of this study are available from the corresponding author upon reasonable request.
