# Supplementary material for: Characteristics of ST11 KPC‐2‐producing carbapenem‐resistant hypervirulent Klebsiella pneumoniae causing nosocomial infection in a Chinese hospital
Source: J Clin Lab Anal. 2022 May 6;36(6):e24476. doi: 10.1002/jcla.24476 (PMC9169163; doi:10.1002/jcla.24476)
Supplement: Supplementary file 1 — Appendix S1 [file JCLA-36-e24476-s002.docx]

Supplementary Material 1: Primers used in this study

| Primer name | DNA sequence (5′‐3′) | Amplicon  size (bp) |
| --- | --- | --- |
| Carbapenemase genes | | |
| *bla*_KPC_ | F: TGTCACTGTATCGCCGTC | 1010 |
|  | R: CTCAGTGCTCTACAGAAAACC |  |
| *bla*_NDM_ | F: ATTAGCCGCTGCATTGAT | 800 |
|  | R: CATGTCGAGATAGGAAGTG |  |
| *bla*_IMP_ | F: ACCGCAGCAGAGTCTTTGCC | 750 |
|  | R: AACCAGTTTTGCCTTACCAT |  |
| *bla*_VIM_ | F: AGTGGTGAGTATCCGACAG | 261 |
|  | R: ATGAAAGTGCGTGGAGAC |  |
| *bla*_OXA-48_ | F: TTGGTGGCATCGATTATCGG | 438 |
|  | R: GAGCACTTCTTTTGTGATGGC |  |
| Other β-lactamase genes | | |
| *bla*_CTX-M-1,3,10~12,15_ | F: TTTCGGAAGCATAAAATCGG | 1021 |
|  | R: GGCGATAAACAAAAACGGAA |  |
| *bla*_CTX-M-2,4~7, Toho-1_ | F: ATGATGACTCAGAGCATTCG | 832 |
|  | R: TCCCGACGGCTTTCCGCCTT |  |
| *bla*_CTX-M-9,13~14,16~19, Toho-2_ | F: AAAAATGATTGAAAGGTGGT | 1242 |
|  | R: GTGAAGAAGGTGTTGCTGAC |  |
| *bla*_SHV_ | F: GGGTTATTCTTATTTGTCGC | 785 |
|  | R: TTAGCGTTGCCAGTGCTC |  |
| *bla*_TEM_ | F: ATAAAATTCTTGAAGACGAAA | 972 |
|  | R: GACAGTTACCAATGCTTAATCA |  |
| Virulence genes | | |
| *iucA* | F: ATAAGGCAGGCAATCCAG | 2927 |
|  | R: TAACGGCGATAAACCTCG |  |
| *iroN* | F: GTCCGGCGGTAACTTCAGCC | 829 |
|  | R: TCAGAATGAAACTACCGCCC |  |
| *_p_rmpA* | F: TACATATGAAGGAGTAGTTAAT | 505 |
|  | R: GAGCCATCTTTCATCAAC |  |
| *_p_rmpA_2_* | F: TGTGCAATAAGGATGTTACATTAGT | 609 |
|  | R: TTTGATGTGCACCATTTTTCA |  |
| *entB* | F: GTCAACTGGGCCTTTGAGCCGTC | 400 |
|  | R: TATGGGCGTAAACGCCGGTGAT |  |
| *irp-1* | F: TGAATCGCGGGTGTCTTATGC | 238 |
|  | R: TCCCTCAATAAAGCCCACGCT |  |
| *irp-2* | F: AAGGATTCGCTGTTACCGGAC | 287 |
|  | R: AAGGATTCGCTGTTACCGGAC |  |
| *fimH* | F: TGCTGCTGGGCTGGTCGATG | 550 |
|  | R: GGGAGGGTGACGGTGACATC |  |
| *mrkD* | F: AAGCTATCGCTGTACTTCCGGCA | 340 |
|  | R: GGCGTTGGCGCTCAGATAGG |  |
| *wabG* | F: ACCATCGGCCATTTGATAGA | 683 |
|  | R: CGGACTGGCAGATCCATATC |  |
| *wcaG* | F: GGTTGGKTCAGCAATCGTA | 169 |
|  | R: ACTATTCCGCCAACTTTTGC |  |
| *allS* | F: CCGAAACATTACGCACCTTT | 1090 |
|  | R: ATCACGAAGAGCCAGGTCAC |  |
| *peg-344* | F: CTTGAAACTATCCCTCCAGTC | 508 |
|  | R: CCAGCGAAAGAATAACCCC |  |
| Capsular type gene | | |
| *wzi* | F: GTGCCGCGAGCGCTTTCTATCTTGGTATTCC | 580 |
|  | R: GAGAGCCACTGGTTCCAGAA[C or T]TT[C or G]ACCGC |  |
| Multilocus sequence typing genes | | |
| *gapA* | F: TGAAATATGACTCCACTCACGG | 680 |
|  | R: CTTCAGAAGCGGCTTTGATGGCTT |  |
| *infB* | F: CTCGCTGCTGGACTATATTCG | 596 |
|  | R: CGCTTTCAGCTCAAGAACTTC |  |
| *mdh* | F: CCCAACTCGCTTCAGGTTCAG | 698 |
|  | R: CCGTTTTTCCCCAGCAGCAG |  |
| *pgI* | F: GAGAAAAACCTGCCTGTACTGCTGGC | 759 |
|  | R: CGCGCCACGCTTTATAGCGGTTAAT |  |
| *phoE* | F: ACCTACCGCAACACCGACTTCTTCGG | 603 |
|  | R: TGATCAGAACTGGTAGGTGAT |  |
| *rpoB* | F: GGCGAAATGGCWGAGAACCA | 755 |
|  | R: GAGTCTTCGAAGTTGTAACC |  |
| *tonB* | F: CTTTATACCTCGGTACATCAGGTT | 686 |
|  | R: ATTCGCCGGCTGRGCRGAGAG |  |
