# Supplementary material for: Characteristics of ST11 KPC‐2‐producing carbapenem‐resistant hypervirulent Klebsiella pneumoniae causing nosocomial infection in a Chinese hospital
Source: J Clin Lab Anal. 2022 May 6;36(6):e24476. doi: 10.1002/jcla.24476 (PMC9169163; doi:10.1002/jcla.24476)
Supplement: Supplementary file 2 — Appendix S2 [file JCLA-36-e24476-s001.docx]

Supplementary Material 2: PFGE dendrogram of CRKP strains causing nosocomial infections


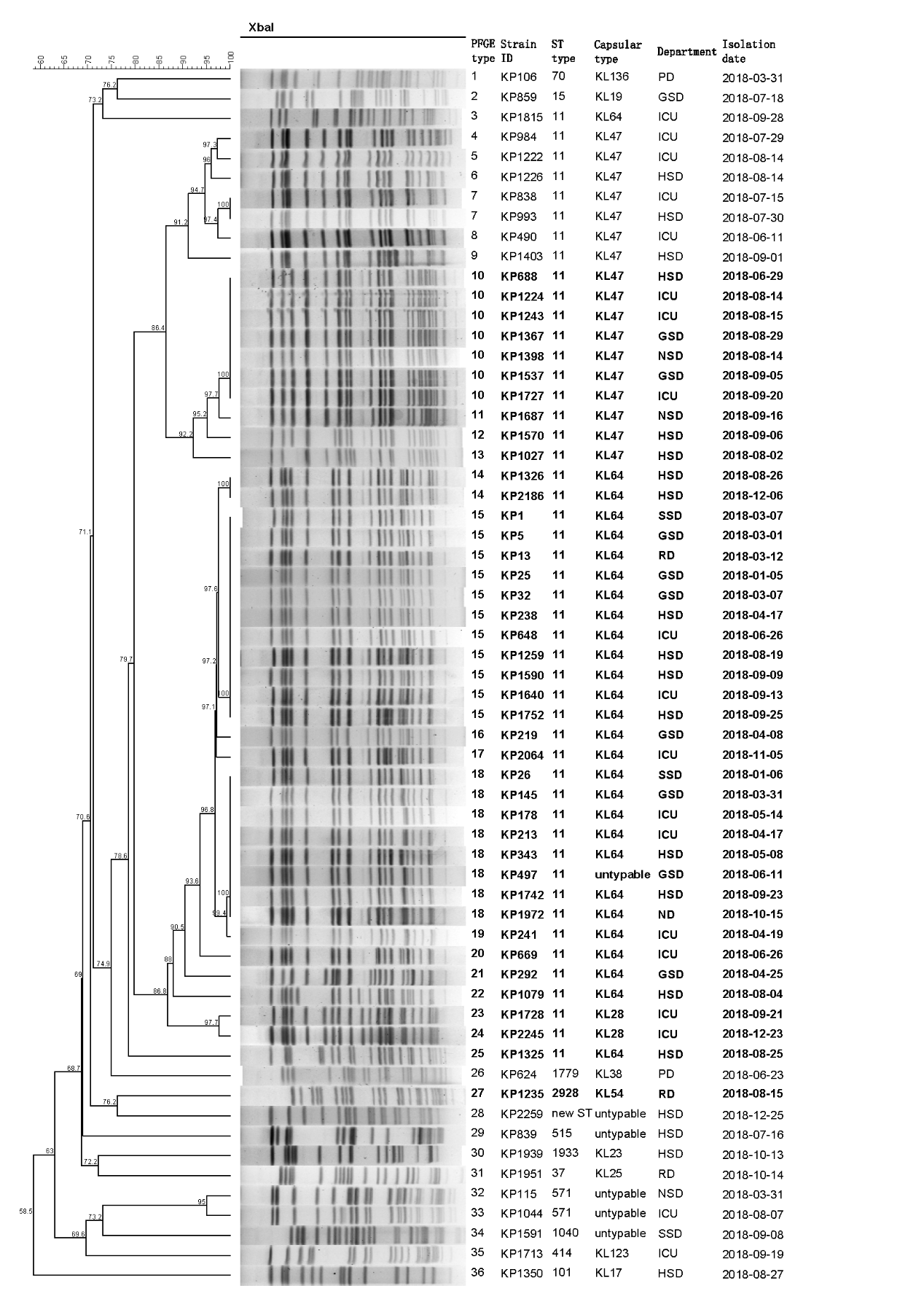


Notes: 41 CR-hvKP strains are indicated by boldface type. PD: Pediatrics Department, GSD: General Surgery Department, ICU: Intensive Care Unit, HSD: Hepatobiliary Surgery Department, NSD: Neurological Surgery Department, SSD: Spinal Surgery Department, RD: Respiratory Department, ND: Nephrology Department.
